# Resilient narratives of a single mother raising a child with autism spectrum disorder: A neurodiversity perspective

**DOI:** 10.4102/ajod.v14i0.1727

**Published:** 2025-10-28

**Authors:** Nettie N. Ndou-Chikwena, Maximus M. Sefotho, Nausheen Ameen

**Affiliations:** 1 Department of Educational Psychology, Faculty of Education, University of Johannesburg, Johannesburg, South Africa

**Keywords:** ASD, single mother, experiences, neurodiversity, resilience, culture, South Africa

## Abstract

**Background:**

Research on experiences of mothers with children living with autism spectrum disorder (ASD) has predominantly focused on psychological distress and caregiving burdens, presenting deficit-centred narratives which inadequately capture other complex narratives of single mothers.

**Objectives:**

This study explored the experiences of a South African single mother, Buhle, in raising a daughter with ASD, focusing on resilience development and acceptance within cultural contexts where ASD and her social status are misunderstood. The study adopts neurodiversity theory and the concept of resilience as its conceptual framework.

**Method:**

Employing an interpretivism paradigm and a single case study research design, data were collected through semi-structured and unstructured interviews. Ethics approval was obtained from the University of Johannesburg. Buhle provided informed consent, allowing her information to be used for research.

**Results:**

Narrative analysis was used to delve deeply into Buhle’s personal and emotional experiences. These narrative themes emerged: navigating the initial trauma of prognosis and diagnosis, building a network of understanding, managing resources without shared responsibilities and transforming challenges to empowerment through social networking, education and advocacy.

**Conclusion:**

The findings challenge deficit narratives by revealing how some single mothers can foster resilience despite significant challenges in raising neurodivergent children in an African cultural context. The South African government’s provision of social and economic support also partly enables resilience.

**Contribution:**

Community-based initiatives should boost public awareness and alleviate the cultural stigma surrounding neurodevelopmental conditions; prioritise resilience, strategy sharing, and advocacy to empower single mothers from survival to empowerment. Public healthcare support services must be improved.

## Introduction

Autism spectrum disorder (ASD) is a complex neurodevelopmental condition characterised by atypical communication, language development, as well as restricted and repetitive behaviour patterns, interests or activities (Johnson 2023). The current body of research focuses on the experiences of parents, families and caregivers of children with ASD and has largely concentrated on aspects such as psychological distress, caregiving burdens and vulnerability (Dira, Machailo & Scholtz 2024; Mazibuko et al. 2020; Ndlovu 2021; Van Niekerk, Stancheva & Smith 2023). Although these studies have yielded significant insights regarding the difficulties encountered by these groups, they often present a one-dimensional narrative that emphasises deficits and struggles over adaptation and personal growth. This ‘deficit’ narrative, as described by Van Der Mark et al. (2019), may unintentionally create a sense of vulnerability and victimisation, which simplifies the complex realities of motherhood. The complex and evolving experiences of single mothers, who often navigate different phases coupled with the social and cultural stigma of being single and of raising a child with ASD, from initial stress and adjustment challenges to the development of resilience, coping strategies and, ultimately, acceptance, are not captured yet. To address this gap, this article presents a case study on the narratives of a single mother, given the pseudonym Buhle, who shares her experiences in caring for her daughter with ASD. Her journey towards resilience and acceptance provides valuable insights into this understudied perspective. The study utilised neurodiversity theory and the concept of resilience as its conceptual framework. This article critically examines current literature on parental, family and caregivers’ experiences in raising children with ASD. Next, we present the study methodology, results, discussions, conclusion and future research directions.

### Conceptualising maternal experiences in raising children with ASD

Research on neurodivergent individuals has gained prominence as a crucial theme in African literature, signalling a broader societal shift towards inclusivity and representation. Among the diverse range of neurodevelopmental conditions, ASD has attracted considerable interest (Kopanska et al. 2021). Literature has covered issues and complexities on diagnosis and identification, support services for children with ASD in educational and health institutions, and experiences of professionals, parents, families and caregivers in caring and supporting children with ASD. Autism spectrum disorder is a multifaceted neurodevelopmental disorder distinguished by challenges in two primary domains: social communication and the presence of restricted or repetitive behaviours and interests (Abebe 2020). Symptoms associated with ASD typically appear in the initial years of a person’s development and can result in significant difficulties in social, occupational or other important aspects of functioning (Johnson 2023). Autism spectrum disorder may also be referred to as a social disability, as individuals affected by it often experience challenges in forming and sustaining relationships with others because of deficiencies in communication and interaction skills (Johnson 2023).

Parenting a child with ASD can be a demanding and stressful endeavour, especially in nations where access to various support services is restricted (Papadopoulos 2021). Challenges are also intensified by factors such as poverty, insufficient resources, limited understanding of the condition (Mbanjwa & Harvey 2023) and cultural factors (Aderinto, Olatunji & Idowu 2023). The burden of care for a child with ASD rests predominantly on the mother, as societal stigma and discrimination surrounding disabilities can lead fathers to neglect their parental responsibilities (Mbanjwa & Harvey 2023). Research indicates that levels of parenting stress and depressive symptoms are notably elevated among mothers (Johnson 2023). Mothers of children with ASD face increased psychological distress, a greater caregiving burden, diminished resilience, and various health-related challenges (Johnson 2023; Papadopoulos 2021). According to Pottas and Pedro (2016), mothers usually dominate as participants in terms of numbers in studies exploring parental or caregivers’ experiences in caring for and supporting children with ASD. For instance, in a systematic review study by DePape and Lindsay (2016), in 31 articles selected for inclusion, 160 fathers and 425 mothers were involved. Furthermore, in a study by van Niekerk, Stancheva and Smith (2023), which aimed at describing the socio-demographic profiles and determining the extent of the burden experienced by caregivers of children and adolescents with ASD, 56 out of 77 participants were mothers.

Reed and Osborne (2019) outline temporal stages experienced by parents of children with ASD, and the experiences of the mother are emphasised. These stages are prognosis, followed by obtaining a diagnosis and contact with healthcare and social service professionals and the final stage of acceptance, coping and resilience (Reed & Osborne 2019). These stages are not fixed at a particular point in time and differ according to environmental factors, cultural factors, and even personality traits. Culture refers to a set of values, beliefs, preferences, and behaviours shared by a group in a particular society, which are handed down from one generation to the next (Mpofu 2024). Cultural beliefs and practices shape attitudes towards ASD in most African contexts (Aderinto et al. 2023). The stigma perpetuated by cultural beliefs views neurodevelopmental conditions as taboo or caused by supernatural factors; there is a myth that such conditions are a form of punishment (Aderinto et al. 2023). Culture also shapes gender norms, which are societal expectations and stereotypes regarding the roles and behaviours considered appropriate for individuals based on their gender (Nartey, Bahar & Nabunya 2023). In African contexts, cultural values, beliefs, and gender norms contribute to the delays in ASD prognosis and diagnosis. A systematic review by Issac et al. (2025) concluded that in low-income countries, cultural values and traditional practices contribute to delayed diagnosis and reduce the accuracy of estimates of ASD prevalence.

In the first phase of ASD prognosis, the mother is the first point of contact; she is the first to notice differences between her child and other children (Grebe et al. 2022). During this period, focus revolves around understanding the nature of the child’s condition, feelings of parental inadequacy and unfit for motherhood (Reed & Osborne 2019). This is followed by the process of obtaining a diagnosis and contact with healthcare and social service professionals (Reed & Osborne 2019). The mother also enters a period of seeking health and social support for herself because of the levels of stress and for the child (Reed & Osborne 2019). She experiences the impact of behavioural problems exhibited by the child and attitudes from their family members and the surrounding community. The final stage is acceptance, coping and resilience.

Resilience is the capacity to adjust or adapt positively to stress or hardship, facilitated by a process that includes both internal and external characteristics (Stein, Hoeft & Richter 2024). Internal attributes consist of personal qualities inherent in resilient individuals. External attributes are the environmental factors that bolster resilience, such as the support provided by family and social networks (Stein et al. 2024). The mother develops ways to cope with the child, such as balancing the needs of the child and her psychological well-being. Organisational patterns, including family routines, flexibility in a family’s day-to-day functionality and planning and the ability to constantly adapt to changes such as family roles, rules and lifestyle, are also a source of resilience (Dürr & Greeff 2020). However, not all mothers get to this final stage. Social and cultural stigma, which manifests in many forms such as negative labelling, rude comments, being blamed for the child’s condition and rejection (McLean & Halstead 2020), hampers the mothers’ transition to this stage. In addition, in developing countries, there are limited resources, such as rehabilitation and care services for mothers with children with special needs to find comfort and support (Singh & Kumar 2022). Hence, the responsibility of the child’s growth falls solely on the shoulders of the mother, and she goes through a vicious cycle of psychological stress and burden with limited support (Singh & Kumar 2022).

There is an emerging body of knowledge in the South African context on the parental, family, mothers’ and caregivers’ experiences in caring for and raising children with ASD, with the majority focusing on general caregiver experiences and challenges (Blumberg 2015; Dira et al. 2024; Fewster & Guruya 2015; Lentoor, Thuli & Maepa 2023; Mazibuko et al. 2020; Ndlovu 2021; Reddy, Fewster & Gurayah 2019; Van Niekerk et al. 2023). Common findings of these studies are significant psychological, emotional and financial burden on caregivers; resource limitations and poor or limited guidance from health professionals; social judgement, isolation and stigma; limited awareness of ASD; and challenges in diagnosis processes. Literature reviews by Phetoe et al. (2023) and Mofokeng et al. (2023) synthesise existing literature on families’ psychosocial experiences and the prevalence of ASD in South African contexts. Research studies by Karrit (2022), Berson and Adams (2024), and Karrit and Coetzee (2025) examine the impact of the coronavirus disease 2019 (COVID-19) pandemic on children with ASD and families. Common findings include disrupted routines causing significant stress, interrupted access to support services and increased parental burden. While these studies capture the experiences of parents, families and caregivers in raising children with ASD, there is an overemphasis on deficit narratives. Literature focuses disproportionately on psychological distress, vulnerability and caregiving burden rather than resilience and acceptance. It is also important to draw attention to two research studies that examine coping strategies and resilience in caregivers of children with ASD. Sumbane (2024) reveals emotion-focused (positive reappraisal, acceptance, and denial) and problem-focused (active coping and peer support) strategies. The study provides a comprehensive examination of both emotion-focused and problem-focused coping strategies. However, there are some elements of a deficit-focused approach in emphasising negative coping strategies such as self-isolation, denial, religion, overprotection and punishment. The study fails to acknowledge the development of resilience over time, particularly how some individuals transition from challenge-focused to strength-based resilience. Fewster and Gurayah (2015) develop a practical roadmap to coping with ASD for practitioners when supporting parents of children with ASD and emphasise the role of practitioners in providing psychosocial support to parents. However, these insights are practitioner-focused rather than exploring the development of parents’ resilience; the study emphasises external support rather than internal resilience building.

There is also limited literature on the experiences of single mothers of children with ASD. The term single parenting describes a scenario where one parent, either the mother or the father, solely undertakes the responsibilities of raising and nurturing children in the absence of the other parent (Ali & Soomar 2019). A single-parent family can be formed through various circumstances, including the death of one parent, divorce or abandonment (Birara 2021). In some cases, a single mother is a woman who has never been married. The woman may set out to have a baby with the intention of raising it alone or involuntarily when she bears a child in the hope that the father will marry her (Birara 2021). Single parenthood is strongly gendered, as most single parents are mothers (Maldonado & Nieuwenhuis 2019). It also appears that the gender of a single parent plays a significant role in shaping social attitudes, with mothers encountering more negative perceptions than fathers. Society tends to view single mothers as individuals who have been unable to sustain a relationship, thus neglecting their obligation to provide a secure family setting for their children (Dor 2021). Additionally, they may be seen as having entered parenthood involuntarily, often because of unexpected pregnancies or unwise personal decisions (Dor 2021). Consequently, single mothers are frequently perceived as unhappier, deviant, troubled, and lacking in effective child-rearing capabilities compared to their counterparts (Dor 2021). On the other hand, the societal perception of single fathers tends to be more positive, as they are regarded as responsible and caring figures in their children’s lives (Dor 2021). This favourable view is often a result of their need to take on parental duties because of situations such as the mother’s passing or her incapacity to fulfil her expected roles (Dor 2021).

In South Africa, a significant number of children are brought up by single parents, who are mostly mothers (Purmasir 2018). According to a survey conducted by the South African Institute of Race Relations (SAIRR) in 2013, merely 33% of children in the country reside with both parents. The findings further indicated that slightly more than 39% of children live solely with their mothers, while only 4% are raised exclusively by their fathers (Purmasir 2018). In the same vein, Statistics South Africa (2024) unveils that most children live with only their mothers because of labour migration of the fathers, as well as low marital rates of mothers. There is a limited body of literature addressing the experiences of single mothers raising children with ASD. Although a study by Purmasir (2018) examined experiences of single parents of children diagnosed with ASD, the study generalised single parents and included single mothers, fathers and grandmothers. We argue that single parenthood is highly gendered, and single parenthood negatively affects mothers more than fathers (Dor 2021; Maldonado & Nieuwenhuis 2019). This represents a critical void, as single mothers face unique experiences they navigate while raising a child with ASD. These complications revolve around financial constraints, the cultural and societal stigma of being a single mother, as well as the difficulties compounded by the myths and stereotypes associated with neurodevelopmental conditions, which may be perceived as a punishment from God (Aderinto et al. 2023). Thus, the intersection of single motherhood and raising a child with ASD within these cultural contexts presents a unique experiential landscape that requires focused academic enquiry.

To our knowledge, a study by Mthimunye (2014) is the sole study that has investigated the experiences and coping mechanisms of six single mothers in a low-income community in the Western Cape province of South Africa. The study unveiled challenges such as a lack of knowledge, inexperience, personal challenges and society’s perceptions of ASD. The support from family and community contributed to resilience development and coping to outweigh the challenges. Unlike Mthimunye’s deficit-focused approach, this study employs a strength-based neurodiversity framework that celebrates neurological differences and explores transformative growth processes. The gap between these two studies allows this research to benefit from contemporary understanding of ASD and a shift from external support dependency to internal resilience development. This study represents the first South African research to examine single motherhood and ASD through a comprehensive neurodiversity resilience lens.

We advance an understanding of Buhle’s experiences by emphasising both the difficulties she encountered and the strengths acquired by concentrating on her story of adaptability and resilience over deficiencies and challenges. The goal of this study is to change the perception of single mothers as victims of life circumstances to proactive, empowered mothers who gain significant psychological resources and skills along the course of their parenting journey.

Merging the two theoretical perspectives, namely, neurodiversity theory and the concept of resilience, establishes a comprehensive viewpoint for exploring the experiences of a single mother raising a child with ASD.

### The neurodiversity theory

The conceptualisation of disability has evolved considerably in the last 50 years, as illustrated by the World Health Organization (WHO) Disability Classification Manuals. The traditional medical model regarded disabilities as personal impairments and dysfunctions (Goldberg 2023). Conversely, the more recent social model has framed disability as the incongruence between an individual’s abilities and needs and the environmental conditions regarding accessibility and accommodation (Goldberg 2023). This shift from the medical to the social model signifies a fundamental change in perspective, viewing disability not merely as an individual issue, but as a construct shaped by societal factors (Goldberg 2023).

Neurodiversity represents a contemporary approach to acknowledging individuals who possess neurodevelopmental variations (Vanderburg, Pagán & Pearson 2024). In the late 1990s, sociologist Judy Singer introduced the term neurodiversity to the academic sphere to present a new perspective on the variations in human perception and communication styles (Goldberg 2023). The framework seeks to transform the perception of neurodiverse conditions across educational, clinical, research and societal contexts (Lerner, Gurba & Gassner 2023). Neurodiversity shifts the focus away from perceived behavioural limitations and underscores that individuals grow and develop in distinct ways (Vanderburg et al. 2024). Diagnoses such as ASD and other conditions are regarded as natural, alternative developmental paths rather than as disabilities (Vanderburg et al. 2024). A lack of alignment between an individual and their environment can lead to detrimental outcomes, including mental health challenges and burnout (Vanderburg et al. 2024). This framework addresses the main weakness of the former two models: failure to view disability from the ‘affected’ person’s perspective.

### The concept of resilience

The concept of resilience is often defined as a personality trait or a combination of traits that reflect an individual’s ability to cope with difficulties and return to a state of stability (Park et al. 2021). It is also perceived as a process or capability that can be improved or learned (Park et al. 2021). The evolution of the concept of ‘resilience’ can be traced back to the 1800s (William 2024). Until the 1950s, it was predominantly examined in psychological literature through the lens of unconscious defence mechanisms (Grygorenko & Naydonova 2023). In the 1960s, the focus transitioned to conscious coping strategies, and by the 1980s, the framework evolved to incorporate protective and risk factors (Grygorenko & Naydonova 2023). Research dissemination in multiple fields of psychology has aimed to ascertain whether resilience is a fixed personality trait or a dynamic state (Grygorenko & Naydonova 2023). The emphasis is placed on identifying the traits of individuals who have thrived and succeeded despite facing significant challenges, such as parenting a neurodivergent child in a cultural environment that stigmatises both social status and neurodevelopmental conditions.

**FIGURE 1:**
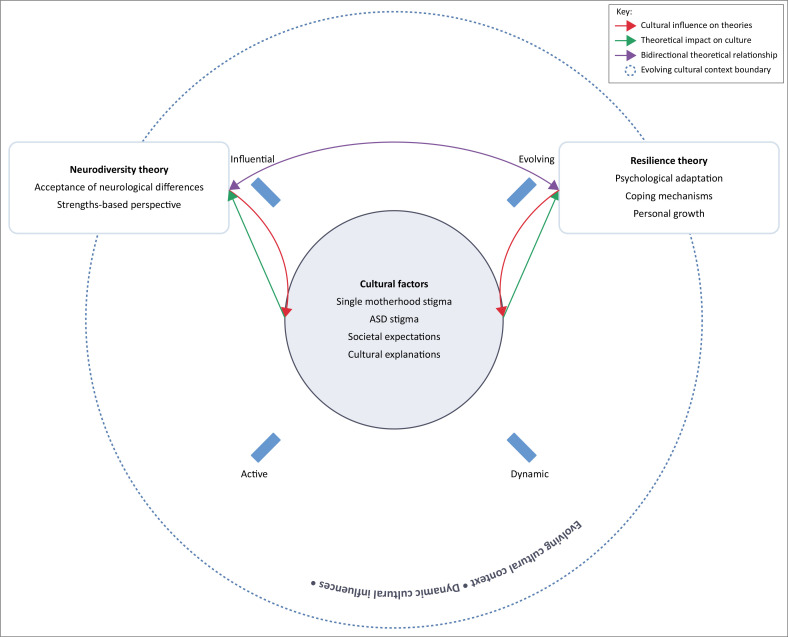
A conceptual framework for understanding single mothers’ experiences in raising children with autism spectrum disorders in African cultural settings.

Figure 1 presents a conceptual framework on how Buhle fosters acceptance and resilience while nurturing her daughter with ASD in an African cultural environment. The framework merges neurodiversity theory, which views ASD as a natural variation and focuses on unique strengths rather than deficits, with the resilience concept, which explores how individuals adjust and succeed through internal psychological assets and coping strategies. The diagram illustrates cultural factors, such as the stigma surrounding single motherhood, the stigma associated with ASD, societal expectations, and cultural interpretations, as central and evolving influences that both shape and are shaped by various theoretical perspectives. Bidirectional arrows illustrate the dynamic interactions between cultural context and theoretical frameworks, emphasising that cultural factors are not static background elements, but rather active forces that perpetually influence the mother’s journey. This comprehensive approach transcends simplistic narratives of vulnerability, emphasising the mother’s agency, adaptability, and personal development, acknowledging her as a proactive individual who cultivates significant psychological resources throughout her parenting experience.

## Research methods and design

### Research paradigm

The selection of a research paradigm influenced the methodologies employed and the results obtained in the study (William 2024). The philosophical underpinnings of this study were based on the interpretive paradigm. The interpretivism paradigm holds the belief that individuals construct knowledge based on their interpretations and experiences. This paradigm used subjective interpretation and constructed new theoretical and social constructs through studying human behaviour and social phenomena (William 2024). Interpretivism enabled capturing in-depth data on the subjective experiences of the single mother in raising her daughter with ASD. Although this paradigm has been questioned on the generalisability of the research findings to other contexts (Pulla & Carter 2018), this study was not concerned about generalisations, as its main thrust was to unveil rich, nuanced and subjective experiences faced by Buhle in caring for and supporting her child with ASD within the South African cultural context.

### Research design

A single case study was adopted for a holistic examination of the issue under study (ed. Salkind 2010). This study deliberately employs this design, as its primary objective is to unveil the rich and subjective experiences faced by Buhle in caring for and supporting her child with ASD (Coombs 2022). Deeply contextualised single cases often reveal the unique and complicated insights that larger samples might overlook. This case was considered distinctive as it illuminated issues of resilience and acceptance, themes that have received limited scholarly attention in research examining maternal experiences of caring for and supporting children with ASD in the South African context.

### Participant selection and data collection techniques

Buhle, a 39-year-old single mother of two, was purposively selected for this single case study from the Centre for Neurodiversity’s database of parents of children with ASD. Her eldest child has been diagnosed with ASD. The selection criteria included being a single mother caring for a child with a formal ASD diagnosis and willingness to share in-depth personal experiences. The participant was contacted and invited to participate through a telephone invitation.

Data were collected through a semi-structured interview and an unstructured interview. Trust and depth were acquired through interviewing the same participant repeatedly (Osborne & Grant-smith 2021). This longitudinal approach allowed for the development of rapport, explorations of emerging narrative themes and member checking in case of misinterpretations. Although interviews tend to be time-consuming, they were utilised to get to the heart of personal experiences and to get a sense of the context of the mother’s worldview and how her experiences contribute to knowledge in conceptualising the maternal experiences in caring for and supporting a child with ASD (Osborne & Grant-smith 2021).

### Data analysis

Narrative analysis enabled researchers to explore Buhle’s personal and emotional experiences as a single mother raising a child with ASD. This approach facilitated an understanding of the psychological, emotional and social experiences she has had (Benson 2018). Narrative analysis functions as a qualitative research approach that emphasises stories as data, facilitating a more profound comprehension of human experiences and identities (Bamberg 2012). Researchers engaged in this form of analysis to derive a variety of interpretations and conclusions that are both meaningful and focused on different aspects (Parcell & Baker 2017). These aspects included how the story is structured, its substance, its function and how it is told (Parcell & Baker 2017). Hence, narrative analysis provided insights into how cultural, social and economic contexts influence the experiences of single mothers raising children with ASD. The narratives were transcribed, then read repeatedly for familiarisation, holistic understanding, and the identification of patterns and themes (Nasheeda et al. 2021).

### Ethical considerations

Ethical considerations were prioritised throughout this research. Ethical clearance to conduct this study was received from the University of Johannesburg Faculty of Education Research Ethics Committee (Sem 2-2020-055). Buhle was contacted and invited to the Centre, where the aims of the research project were thoroughly explained. Buhle provided informed consent, allowing the researchers to use her information for research purposes. Voluntary participation was also strongly emphasised in the consent form. A pseudonym was used to ensure confidentiality and anonymity, and the data were stored securely. Recognising the potential vulnerabilities of single mothers raising children with ASD, interviewers avoided judgemental language, built rapport to ensure the participant felt at ease and maintained follow-up contact to support her well-being. This research was conducted through a neurodiversity-affirming lens, recognising her expertise in the issue under study and avoiding pathologising language about ASD or other conditions she disclosed.

## Results of the study

The following narrative themes emerged from the study: navigating the initial trauma of prognosis and diagnosis, building a network of understanding, managing resources without shared responsibilities and transforming challenge into empowerment. These themes align with recognised stages of disability acceptance in the literature from Reed and Osborne (2019), while illustrating Buhle’s experiences, from the initial prognosis and diagnosis through the development of resilience and acceptance. The findings highlight financial, cultural and psychosocial challenges faced by Buhle in raising a child with ASD, contributing nuanced insights to our understanding of this process.

### A brief profile of Buhle

Buhle is a 39-year-old mother of two living in an underserved community in Soweto, Johannesburg. Her older child, Phatie, is 13 years old and has ASD. Buhle lives with her diabetic mother and also manages her own diabetes as well. After losing her permanent job following Phatie’s birth, Buhle now depends on part-time work to support her family. The household income is further supported by government grants: an old-age grant for Buhle’s mother and a disability grant for Phatie, both providing vital financial assistance for the family.

### Navigating the initial trauma of prognosis and diagnosis

Buhle’s current life experiences stem from a series of traumatic events surrounding Phatie’s birth and diagnosis. Buhle experienced significant psychological shock and stress during the prognosis and diagnosis, compounded by medical complications during pregnancy and birth that led to her diabetes diagnosis. The initial prognosis was psychologically devastating, and she had to resign from her job. She also highlighted that the diagnosis had something to do with cultural factors. The following are her sentiments:

‘I had a complicated pregnancy, which resulted in me being in high care. I was told that I need to proceed with the c-section labour as I lost water in the 7 month of pregnancy. I was diagnosed with diabetes while I was pregnant. You know, we tend to ignore such issues, and we think it has something to do with cultural things when the child is diagnosed with such conditions … My baby was not diagnosed immediately after birth, but I was told that she wasn’t going to manage to function well. She spent two weeks in the ICU. When they discharged her, they told me that my child was blind. So, I was told to go for therapy, yoh! She was seen by many specialists … occupational therapy … ah, I did a lot of people. They told me that my baby was going to be a potato coach [*sic*], if you understand … she was a child who was not going to do anything. She will struggle to walk and talk … So, because I was working at that time, I had to resign, so that I could take care of her accordingly. I was not supposed to put her on my back, as we African women carry our babies. I had to handle her with care because of her fragile bones. So, along the way, she developed, and she started to sit. She was premature, remember. Her eyesight was restored. I can’t tell when her eyesight was restored, but it was during the therapy processes and sessions. It was the first thing she recovered from.’

From her narrations, Buhle was not well informed on the nature and characteristics of ASD. When she goes for appointments to meet specialists, she avoids asking a lot of questions because of long queues at the public hospitals:

‘… so, it goes with levels, right? I was told she is at level 3. Now please don’t get me wrong, am afraid to speak to specialists during appointments, because I wouldn’t want to take their time because there will be a long queue outside at the hospitals … and sometimes I feel you know autistic children become so unsettled in a queue so am very grateful for this opportunity to ask questions freely …’

### Building a network of understanding

Her journey to establish psychosocial support was marked by limited family support, cultural misattributions and a single friendship as a cornerstone:

‘To tell the truth, I had only one person in my life at that time, my childhood friend. But I’m not sure if she was supportive, or maybe she was just intrigued by the experience I went through. Maybe she had no choice but to support me. She had no children at that time, but she was my backbone. She would check up on me, my well-being.’

‘So, when it comes to family, everybody was afraid. You know I cannot blame them, because when such things happen in a family, no one knows how to assist. So, they would assume that it’s a cultural thing, and I had to present the child to her father’s family. You know they will come up with all those explanations. But I would just tell them, only God knows about this. I didn’t get the support from them; it’s not like I blame them, I do understand them. So, I experienced all this with the child I could have terminated, you know … because of complications during pregnancy.’

In time, she secured backing from her family and community, largely attributable to her proactive efforts in involving the community in her child’s life. She made efforts to connect her daughter with neighbours and host social gatherings to facilitate interactions with other local children. This engaged approach shifted community attitudes and established a supportive social atmosphere for her daughter. Additionally, she regularly attends meetings and educational forums focused on ASD, showcasing her persistent commitment to education and advocacy:

‘So, when it comes to the community, they only knew about my child when she started to walk. I made a huge birthday party for her, of course, I am not a birthday party person. But I had to do it because I was celebrating her life and achieving milestones. I never thought she was going to walk. She went to a mainstream preschool. I had to explain to the school that my child has a condition. So, she started to walk when she was in pre-school. So, it was a huge thing for me. So, at the party, I narrated my journey to the community so that they would understand my celebration.’

‘I try to engage the community so much. I take all the kids around my area, ask permission from their parents, and we go play. I organise kids’ functions and ask the parents to contribute. This will make other kids understand my child.’

‘… but I am learning, everywhere, where there is autism am there everywhere. I am willing to learn. Even while I was working, during Children’s Day, they would invite an Occupational Therapist to inform us about Autism, even though I am the only one with an Autistic child. I am learning …’

### Managing resources without shared responsibilities

It has been observed that Buhle’s pregnancy and the subsequent birth affected her ability to maintain full-time employment, later leading her to forfeit her career to care for her child. As a single parent, she is tasked with managing all responsibilities independently. She utilises public resources to aid in supporting her child and participates in various part-time employment opportunities:

‘I must handle all my own; the father is not in the picture. You know what, I also thank the therapists and doctors … they assisted me in receiving the disability grant, even though I was working at that time … But now I am no longer working full-time, I do part-time jobs here and there.’

‘My baby is enrolled at a special government school, and I do not pay fees. I only pay for her transport. At her school, the occupational therapists are there, and nurses are there for support.’

### Transforming challenge into empowerment

Her development of resilience and acceptance is evident through celebration of her daughter’s developmental milestones, continuous learning, recognition of parental acceptance as key, providing a holistic approach to the child’s development and breaking cultural taboos. ‘*Sehole ho ‘Ma-Sona, se setle*’: this is a Sesotho proverb meaning that a child with a disability is beautiful in his or her mother’s eyes (Sefotho 2024). As she was narrating stories about her daughter, one could see a glow and acceptance in her eyes. She is driven by the need to make people understand the nature and characteristics of her daughter’s condition and accommodate her:

‘Yeah, I am a mother; I must be strong for her. I had to inform my community to understand and accept my child. So, they interact with her so well. So, I don’t have a problem if she is playing outside, because I know she is safe. Everyone knows about her. They help her, even crossing the road. They assist.’

‘… in my community, I have made them aware of my child because she is very friendly and they didn’t understand things like that … so now in my street, every house knows her and when she knocks, they now accommodate her.’

‘You know it starts with you as a parent. As soon as you accept the situation. Better for the child. You will manage to tell the truth. You know these conditions are taboo to talk about. But if you seek help and support for the child, it will be fine. All children have a disability. It’s about you as a parent to understand the child’s emotions and needs. I know her, I just must control her unwanted behaviour, her anger. I know what triggers her happiness. You just learn your child.’

‘So, I started to understand. As you see me at these meetings at the centre, I am trying to learn more about the condition so I can support her better … me and her talk about everything … even about her reproductive health … she has started her monthly periods now … which means she is normal. I do my best to make her happy.’

## Discussion

Four narrative themes emerged from the study: navigating the initial trauma of prognosis and diagnosis, building a network of understanding, managing resources without shared responsibilities, and transforming challenge to empowerment. The results present the complex experiences of adaptation and resilience experienced by Buhle, a single mother raising a daughter with ASD in a context where ASD and her social status are often misunderstood and stigmatised. These findings align with the conceptual framework of the study, which merges neurodiversity and resilience perspectives. The conceptualisation of disability has evolved from a medical model of ‘impairment’ to a social model that recognises environmental factors, with neurodiversity representing a contemporary approach that values neurological variations as natural developmental paths. Despite significant challenges such as her health conditions, cultural and societal perceptions on ASD and on being a single mother, and economic and financial struggles, she proactively develops strategies to access resources, build community support and provide quality care for her child. This demonstrates the resilience theory component of the study’s conceptual framework, which examines how individuals adjust and flourish despite adversity by developing psychological assets and coping strategies. Her experience suggests that the experiences of mothers raising children with ASD are not only portraying them as vulnerable and passive recipients of life struggles, as shown in most research studies, but also as strong individuals who strive towards acceptance and empowerment.

The process of diagnosing ASD is commonly characterised by its long duration, high costs, and significant stress (Manono & Clasquin-Johnson 2023). Perlman and Howe (2022) explain that the prognosis and diagnosis phases are characterised by confusion, feelings of self-blame and severe stress. Cultural contexts may intensify these challenges (Papadopoulos 2021). Aderinto et al. (2023) argue that in African contexts, the diagnosis of ASD is viewed as a punishment or curse. Autism spectrum disorder is viewed as taboo and an issue which cannot be discussed in public spaces. Hence, mothers in such a situation go through mental struggles full of self-blame and feelings of being unfit for motherhood. Sadiki (2023) purports that parenting a child with conditions such as ASD adversely impacts one’s well-being because of societal stereotypes, prejudices, stigma, psychological health struggles, economic struggles and a lack of family support.

The situation is further worsened by the status of being a single mother. Being a single mother results from a variety of life circumstances and influences, including the death of a spouse, divorce, unplanned pregnancies, broken promises or the choice of the mother to raise a child as a single parent (Jacobs & Andrews 2021). However, in an African context, marriage and motherhood are still ascribed adult statuses to women and provide a source of prestige (Ntoimo & Mutanda 2021). Single-mothered families are often viewed through a ‘deficit’ perspective (Jacobs & Andrews 2021). Dor (2021) argues that a single mother is frequently perceived as unhappier, deviant, troubled, and lacking in effective child-rearing capabilities and unable to maintain a family structure. Thus, bearing a ‘fatherless’ child with ASD is given spiritual explanations that the ancestors are not happy; hence, the mother is cursed, or the ancestors of the child are not happy, as the child has not been introduced to the father’s family.

The mother may also feel overwhelmed by the demands of meeting financial obligations, maintaining the household and working (Baluyot et al. 2023). Raising a neurodivergent child can affect the career paths of a mother. The need for intensive and special care for the child may disrupt the parent’s career and full-time employment (Ozdemir & Koç 2023), resulting in opting for self- or part-time employment (Baluyot et al. 2023). However, such work options always come with challenges to meet most financial obligations, and in countries such as Zimbabwe, where there are limited public social grants and adequate free services to cushion individuals from such economic challenges, single mothers in such contexts are in dire situations (Matsai & Raniga 2021). Fortunately, the social grant system in South Africa supports individuals in need of social and economic support (Chagunda 2019). This support is in the form of cash, such as the care dependency grant, child support grant, or disability grant. The support can also be in the form of free access to education and health services for children. This helps cushion single mothers who might face financial challenges in raising their children.

In the context of interlinked challenges faced by single mothers, it takes resilient traits to overcome such life circumstances and accept that neurodiverse conditions are natural variations rather than viewing them as a deficit that needs to be fixed (Sharma, Chauhan & Gupta 2025) or a curse. Resilience is commonly understood as a dynamic process that facilitates positive adaptation to the environment. This concept includes the collective influence of personal attitudes, beliefs, and skills, which enable individuals to achieve success when confronted with adversity (Ghanouni & Eves 2023). Buhle develops resilience over time, transitioning from deficit and challenge-focused to positivity and acceptance. This transition enabled her to handle parental obligations financially and initiate the involvement of society in raising her child.

Societal stigma on disabilities stems from limited understanding or fear of differences (Olasehinde 2024). However, it takes positive resilience for a mother to bridge the gap between her and society through building social networks and educating the community about such conditions. This creates a supportive and welcoming social structure for the child. Social networks refer to relationships and connections individuals have with family, friends, community, healthcare providers and community organisations (Teslim 2024). Adopting proactive strategies fosters resilience among mothers, enhancing their capacity to adapt to and manage overwhelming demands of care giving and support. Social networks provide various forms of support such as emotional comfort, practical help, and access to information and resources (Teslim 2024).

Much of the existing literature regarding mothers’ experiences in caring for and supporting children with ASD tends to emphasise the adverse effects, such as caregiving burden, unmet needs, negative emotions related to their child, depression, anger, fatigue, and insufficient social support. This ‘deficit’ or ‘misery’ narrative, as described by Van Der Mark et al. (2019), may inadvertently create a sense of vulnerability and victimisation, which simplifies the complex realities of motherhood. Such a viewpoint obscures the resilience and acceptance which other mothers demonstrate. In some cases, mothers have bravely transitioned from the phases of prognosis and diagnosis to a position of empowerment and advocacy, actively reconstructing their identities as resourceful agents rather than passive victims of adversity. They are instrumental in creating meaning and establishing supportive communities amid challenging circumstances.

### Study limitations

This study has a limitation that must be acknowledged. The study focused on a single mother’s experience; therefore, these findings cannot be generalised to represent all single mothers raising children with ASD in South Africa. The participant’s level of education, race, access to resources and personal support networks may have influenced her journey in ways that might not apply to all single mothers. Cultural contexts, socioeconomic status and individual resilience factors vary across different communities.

## Conclusion and recommendations

This research adds to the expanding literature that shifts focus from deficit narratives to emphasise the resilience and acceptance cultivated by some single mothers who are raising children with ASD. The findings reveal that, despite facing significant hurdles, including health complications, cultural stigma, financial difficulties and insufficient support networks, such mothers develop exceptional adaptive abilities and transform their challenges into empowering experiences. The South African government is also commended for its provision of social and economic assistance through social grants, as well as for ensuring free access to educational and healthcare services. Such services act as a buffer for those who require support.

However, we advocate for structural improvements within the healthcare system to alleviate the prolonged wait times that currently detract from the quality of care. The issue of lengthy queues hampers the effectiveness of interactions between professionals and clients, as some clients may refrain from asking essential questions because of the presence of others waiting. By increasing healthcare staffing and utilising technology for virtual consultations, the client experience could be transformed from hurried interactions to more substantial engagements. Furthermore, there is a need to establish more community-based support groups for mothers of neurodivergent children. These groups could also participate in campaigns aimed at combating the stigmatisation of neurodevelopmental conditions and addressing the social status of various groups of women within communities. For future research, we suggest conducting longitudinal studies that monitor the development of resilience among diverse groups of mothers who care for and support children with such conditions across various socio-economic and cultural backgrounds.
